# Correlation Between Food Habits and Mental Disorders in the Adult Population of São Paulo City, Brazil: A Cross‐Sectional Study

**DOI:** 10.1002/hsr2.71389

**Published:** 2025-11-16

**Authors:** Jéssica Leitão Morilla, Luiz Henrique da Silva Nali, Fernanda Simões da Costa Fujino, Daniel Ramos Olcerenko, Patrícia Colombo‐Souza

**Affiliations:** ^1^ Medical School, Santo Amaro University São Paulo Brazil; ^2^ Graduate Program in Health Sciences Santo Amaro University São Paulo Brazil

**Keywords:** diet, eating behavior, mental disorders, nutritional status

## Abstract

**Background and Aims:**

Understanding the prevalence of mental disorders and associated factors is essential for public health. This study examined the associations between sociodemographic characteristics, dietary patterns, and common mental disorders among adults in São Paulo, Brazil, during the COVID‐19 pandemic.

**Methods:**

A cross‐sectional study was conducted with 470 adults recruited online. Mental disorders were assessed using the SRQ‐20 (cut‐off ≥ 7), and dietary intake was evaluated with a Food Frequency Questionnaire (FFQ) that captured both frequency and portion sizes. Sociodemographic data were collected via a self‐reported survey. Data analysis employed *χ* ^2^ tests, independent *t*‐tests, and Pearson correlations, with a significance level of *p* < 0.05.

**Results:**

A higher prevalence of mental disorders was observed among younger adults (18–30 years), single individuals, those with secondary education, and those receiving psychotherapeutic care (all *p* < 0.001). Dietary analysis revealed that individuals with disorders consumed significantly fewer vegetables (*p* < 0.001) but more fruits (*p* < 0.001) and oils/fats (*p* < 0.05) compared to those without disorders. No significant difference was found in processed meat consumption (*p* = 0.35). Correlation analyses revealed a positive association between adequate oil/fat consumption and SRQ‐20 scores (*r* = 0.11; *p* < 0.01). Although inadequate meat consumption showed a statistically significant association (*p* = 0.004), the correlation coefficient was negligible (*r* ≈ 0.00).

**Conclusion:**

This study reveals significant associations between sociodemographic factors, dietary patterns, and mental disorders during the pandemic. Individuals with disorders exhibited a distinct dietary profile characterized by lower vegetable intake but higher consumption of fruits and fats. The correlation findings highlight the need for cautious interpretation of statistically significant but clinically negligible associations.

## 1 Introduction

It is crucial to know the prevalence of mental disorders such as depression, anxiety, alcoholism, psychosis, and epilepsy in the communities, as well as to recognize the associated factors. These psychopathological disorders usually become disabling for the individuals who suffer from them. Sociodemographic factors influence their appearance [1].

Depression is a public health challenge and a multifactorial disorder. It arises from complex interactions among social, psychological, biological, and genetic factors [2]. Symptoms affect mood, thinking, movement, and body, and reduce the quality of life and increase suicide risk [3]. Depression has a significant global burden; in Brazil in 2019, 10.2% of adults were diagnosed with depression [4]. The prevalence was higher among women and residents of Southeast Asia (11.5%), indicating demographic differences [5].

Emerging evidence posits that the pathophysiology of depression involves dysregulation of monoaminergic neurotransmission, neurotrophic factors, circadian rhythms, oxidative stress, inflammatory pathways, and epigenetic modifications [6]. Notably, neurotransmitter imbalances, such as serotonin (5‐HT) deficiency, have been linked to inadequate dietary patterns, as these biomolecules depend on both endogenous synthesis and nutrient availability from food sources [3]. Nutritional psychiatry research has thus focused on the role of micronutrients and macronutrients—including folate, omega‐3 and omega‐6 fatty acids, vitamins B6, B12, and D, zinc, magnesium, and tryptophan—in modulating neurobiological processes implicated in mood regulation [3, 7, 8, 9]. Omega‐3 fatty acids, found in fish, and omega‐6 fatty acids, found in vegetable oils, support brain cell membranes and help regulate inflammation. Tryptophan, found in foods such as brown rice, beans, and beef, helps produce 5‐HT. B vitamins and zinc aid neurotransmitter production and nerve cell survival. Vitamin D, produced with sunlight, affects hormone and nerve function [3, 10].

Observational studies suggest that adherence to nutrient‐dense dietary patterns, such as the Mediterranean diet, is correlated with reduced depressive symptomatology. A longitudinal UK study reported a significant decline in depressive symptoms over 5 years among individuals consuming high amounts of vegetables, fruits, and fish [11]. This finding was corroborated by Italian research [8]. Conversely, diets rich in refined carbohydrates and low in fiber—associated with pro‐inflammatory states—may exacerbate depression risk [7, 12]. Yin et al. [7] demonstrated a 17% reduction in depressive symptoms after 3 years of Mediterranean diet adherence, while Gopinath et al. [12] identified elevated glycemic intake and reduced vegetable consumption among individuals with depressive symptoms. These findings align with hypotheses linking chronic inflammation to mood disorders, as evidenced by Adjibade et al. [13], who proposed anti‐inflammatory diets as preventive strategies, particularly in sedentary populations.

São Paulo, Brazil's largest and most populous city, presents an ideal setting for this investigation due to its diverse socioeconomic and cultural composition, which influences dietary habits. As a major urban center, São Paulo exhibits significant variability in food accessibility, dietary behaviors, and mental health outcomes, making it a critical location for examining the nexus between diet and mental disorders. Therefore, this study aimed to systematically examine the associations between dietary patterns and mental disorders among adults residing in São Paulo, Brazil.

## 2 Methods

### 2.1 Study Design and Setting

This cross‐sectional observational study was conducted among adults residing in São Paulo, Brazil. The study adhered to the STROBE (Strengthening the Reporting of Observational Studies in Epidemiology) guidelines for reporting observational research.

### 2.2 Participants

This cross‐sectional study recruited a convenience sample of adults aged 18 years or older residing in São Paulo, Brazil. The recruitment and data collection were conducted online between September 2020 and April 2021 via social media platforms, community networks, and email chains. This approach was adopted to ensure participant access and comply with social distancing measures during the COVID‐19 pandemic, while also aiming to include residents from all city regions. The inclusion criterion was being a resident of São Paulo; participation was voluntary, and no specific exclusion criteria were defined. The questionnaire was self‐administered. For the present analysis, only responses with complete data for both diet and depression variables were included.

It is important to acknowledge that the convenience sampling method introduces a potential for selection bias. Participants were self‐selected, likely leading to an overrepresentation of individuals with greater access to digital platforms, higher literacy levels, and a pre‐existing interest in the study's themes (health and diet). Consequently, the results may not be fully generalizable to the entire adult population of São Paulo, particularly to groups with limited internet access or lower socioeconomic status. These limitations are common in web‐based surveys, particularly during the pandemic, and should be considered when interpreting the findings.

### 2.3 Variables and Data Sources

Sociodemographic Characteristics: Assessed using a 7‐item self‐reported questionnaire covering age, gender, income, education, and other socioeconomic factors.

Mental disorders were screened with the Brazilian Self‐Reporting Questionnaire (SRQ‐20), a 20‐question tool for nonpsychotic disorders. Each “yes” answer scores 1 point (0–20 total). Following Brazilian studies (with 83% sensitivity and 81% specificity), scores of 7 or higher indicate probable mental disorders, as recommended by the WHO for community studies [14, 15].

Dietary intake was assessed using a structured Food Frequency Questionnaire (FFQ) adapted from a validated instrument [16]. The questionnaire captured the weekly consumption frequency (times/week) and the typical daily portion sizes for 10 predefined food groups: cereals and pasta, vegetables, fruits, legumes, meats, processed meats, dairy products, sweets, oils and fats, and alcoholic beverages. Based on this data, the consumption of each food group was classified as “adequate” or “inadequate” according to the quantitative recommendations (number of daily servings) established by the Dietary Guidelines for the Brazilian Population [17]. For example, adequate vegetable consumption was defined as the intake of at least five servings per day, as recommended by health authorities. This method allowed for a direct comparison of reported consumption with national guidelines, moving beyond mere frequency to incorporate portion size.

The FFQ was administered electronically, and participants self‐reported their responses through an online platform. Only completed questionnaires were included in the final analysis to ensure data quality, and all collected information was anonymized to maintain confidentiality.

### 2.4 Data Collection and Management

Participants provided electronic informed consent before completing the questionnaires. Data were anonymized to protect confidentiality, and researchers signed confidentiality agreements. The study was approved by the Research Ethics Committee (Protocol No. 4.090.976).

### 2.5 Statistical Analysis

Descriptive statistics presented frequencies and proportions for categorical variables and means ± standard deviations for continuous variables. Three primary bivariate analyses were conducted. First, the *χ* ^2^ test assessed differences in sociodemographic characteristics between adults with (SRQ‐20 ≥ 7) and without common mental disorders. Second, the independent samples *t*‐test was used to compare the weekly consumption frequencies of food groups between these two groups. Third, Pearson's correlation coefficient was used to test the linear association between continuous SRQ‐20 scores and consumption scores for each food group.

The assumptions for each test were verified, including normality of distribution using the Shapiro–Wilk test and homogeneity of variances using Levene's test for the *t*‐tests. A two‐tailed *p* value < 0.05 was considered statistically significant. All analyses were performed using IBM SPSS Statistics (Version 28.0; IBM Corp., 2021).

### 2.6 Bias Considerations

Potential biases included self‐reporting errors (e.g., dietary recall inaccuracies) and selection bias due to online recruitment, which may underrepresent populations with limited internet access. The use of validated instruments (SRQ‐20, QFCA) was aimed at mitigating measurement bias.

### 2.7 Ethical Compliance

The study adhered to ethical principles outlined in the Declaration of Helsinki. Participant anonymity and data security were rigorously maintained throughout the study. All volunteers have signed a written consent form, and the researchers have agreed and signed a Confidentiality Term. The project was approved by the Research Ethics Committee, under protocol n° 4.090.976.

## 3 Results

The sampling population characteristics and sociodemographic findings are described in Table 1. The Chi‐square test revealed significant differences (*p* < 0.001) in the distribution by age groups, with a higher prevalence of common mental disorders among young adults aged 18–30 years (67.7%) compared to those over 60 years (17.5%). Significant differences were also observed in marital status, with a higher prevalence among single individuals (61.7%) and all widowed individuals (100.0%, although representing a small subgroup, *n* = 9), and a lower prevalence among married participants (37.1%). Regarding schooling, the prevalence was higher among those with secondary education (63.1%) compared to those with a bachelor's degree (44.7%). A significantly higher prevalence was observed among individuals receiving mental health care (83.9%) compared to those who were not (40.1%).

**Table 1:** Sociodemographic characteristics of adults from São Paulo, according to the presence of common mental disorders (SRQ‐20).

Sociodemographic characteristic	Total sample (*N* = 470)	SRQ‐20 positive (*n* = 240)	SRQ‐20 negative (*n* = 230)	
	*n* (%)	*n* (%)	*n* (%)	*p* value
Sex				0.08
Male	120 (25.5)	53 (44.2)	67 (55.8)	
Female	350 (74.5)	187 (53.4)	163 (46.6)	
Age group (years)				< 0.001*
18–30	220 (46.8)	149 (67.7)	71 (32.3)	
31–40	76 (16.2)	36 (47.4)	40 (52.6)	
41–50	76 (16.2)	34 (44.7)	42 (55.3)	
51–60	58 (12.3)	14 (24.1)	44 (75.9)	
60+	40 (8.5)	7 (17.5)	33 (82.5)	
São Paulo region				0.06
South	246 (52.3)	130 (52.8)	116 (47.2)	
West	77 (16.4)	33 (42.9)	44 (57.1)	
North	54 (11.5)	31 (57.4)	23 (42.6)	
East	65 (13.8)	27 (41.5)	38 (58.5)	
Central	28 (6.0)	19 (67.9)	9 (32.1)	
Marital status				< 0.001*
Single	261 (55.5)	161 (61.7)	100 (38.3)	
Married	159 (33.8)	59 (37.1)	100 (62.9)	
Divorced	41 (8.7)	20 (48.8)	21 (51.2)	
Widowed	9 (1.9)	9 (100.0)	0 (0.0)	
Education level				< 0.001*
Primary education	9 (1.9)	3 (33.3)	6 (66.7)	
Secondary education	168 (35.7)	106 (63.1)	62 (36.9)	
Bachelor's degree	293 (62.3)	131 (44.7)	162 (55.3)	
Ethnicity				0.25
White	375 (79.8)	185 (49.3)	190 (50.7)	
Black	75 (16.0)	42 (56.0)	33 (44.0)	
Asian	20 (4.3)	13 (65.0)	7 (35.0)	
Mental health care				< 0.001*
Yes	118 (25.1)	99 (83.9)	19 (16.1)	
No	352 (74.9)	141 (40.1)	211 (59.9)	
Physical activity				0.32
Yes	300 (63.8)	148 (49.3)	152 (50.7)	
No	170 (36.2)	92 (54.1)	78 (45.9)	

* Data presented as *n* (%). *p* values were calculated using the *χ* ^2^ test. A *p* value < 0.05 was considered statistically significant. Percentages are calculated by row for each characteristic (e.g., 44.2% of men were SRQ‐20 positive).

The eating habits of the participants were analyzed using the Food Frequency Questionnaire (FFQ), and the findings are described in Table 2. Food consumption comparisons using an independent *t*‐test revealed that individuals with common mental disorders had a significantly lower intake of vegetables (7.7 ± 5.1 vs. 11.4 ± 5.0; *p* < 0.001) but a significantly higher consumption of fruits (12.3 ± 6.7 vs. 5.7 ± 3.4; *p* < 0.001) compared to those without disorders. A significantly higher consumption of oils and fats was also observed in the group with disorders (12.5 ± 6.1 vs. 11.4 ± 5.3; *p* < 0.05). No significant differences were observed in the consumption of processed meats (1.9 ± 2.1 vs. 1.8 ± 2.1; *p* = 0.35) or other food groups.

**Table 2:** Association between food group consumption frequency and common mental disorders (SRQ‐20 ≥ 7) in adults from São Paulo, Brazil.

Food group	SRQ‐20 positive (*n* = 240)		SRQ‐20 negative (*n *= 230)		*p* value
	Mean ± SD	Median	Mean ± SD	Median	
Cereals and pasta	15.0 ± 8.7	15.0	15.1 ± 8.3	15.0	0.81
Vegetables	7.7 ± 5.1	6.0	11.4 ± 5.0	11.0	**< 0.001**
Fruits	12.3 ± 6.7	10.0	5.7 ± 3.4	5.0	**< 0.001**
Leguminous	7.7 ± 5.2	6.0	8.3 ± 5.2	8.0	0.24
Meat	12.3 ± 6.7	10.0	13.1 ± 5.6	13.0	0.16
Processed meat	1.9 ± 2.1	1.0	1.8 ± 2.1	1.0	0.35
Sweets	14.8 ± 8.6	13.0	13.5 ± 8.0	12.0	0.09
Oils and fats	12.5 ± 6.1	12.0	11.4 ± 5.3	12.0	**0.03**
Milk and dairy	7.3 ± 4.1	7.0	7.5 ± 4.4	7.0	0.65
Alcohol	1.7 ± 2.1	1.0	1.5 ± 1.9	1.0	0.58

*Note:* Data presented as mean ± standard deviation and median. *p* values were calculated using the independent samples *t*‐test (for means). A *p* value < 0.05 was considered statistically significant and is highlighted in bold.

Abbreviation: SD, standard deviation.

Pearson correlations between food consumption adequacy and SRQ‐20 scores Table 3. revealed a significant positive association for adequate consumption of oils and fats (*r* = 0.11; *p* < 0.01). For inadequate meat consumption, a significant association was found (*p* = 0.004), although the correlation coefficient was negligible (*r* = −0.00). No other significant correlations were observed.

**Table 3:** Correlation between consumption scores of food groups and SRQ‐20 scores among adults in São Paulo, Brazil.

Food group	*n*	Mean SRQ‐20 ± SD	Pearson's *r*	*p* value
Cereals and pasta				
Adequate	83	7.8 ± 4.3	−0.03	0.78
Inadequate	387	7.3 ± 4.2	−0.06	0.21
Vegetables				
Adequate	86	7.4 ± 4.4	−0.14	0.36
Inadequate	384	7.4 ± 4.2	0.01	0.81
Fruits				
Adequate	85	7.1 ± 4.4	−0.20	0.58
Inadequate	385	7.4 ± 4.2	−0.08	0.10
Legumes				
Adequate	200	7.0 ± 4.4	−0.04	0.16
Inadequate	270	7.7 ± 4.2	0.04	0.16
Meat				
Adequate	264	6.9 ± 3.8	−0.12	0.65
Inadequate	206	8.1 ± 4.5	−0.00	**0.004**
Processed meats				
Adequate	343	7.3 ± 4.2	−0.03	0.50
Inadequate	127	7.6 ± 4.3	0.00	0.95
Sweets				
Adequate	280	7.1 ± 4.1	−0.10	0.53
Inadequate	190	7.7 ± 4.5	0.10	0.13
Oils and fats				
Adequate	447	7.3 ± 4.2	0.11	**< 0.01**
Inadequate	23	7.3 ± 4.2	0.09	0.66
Milk and dairy				
Adequate	71	6.8 ± 4.3	−0.17	0.43
Inadequate	399	7.5 ± 4.2	0.00	0.89
Alcohol				
Adequate	460	7.3 ± 4.2	0.08	0.08
Inadequate	10	9.1 ± 3.6	0.10	0.76

*Note: p* value were calculated using Pearson's correlation test. A *p* value < 0.05 was considered statistically significant and is highlighted in bold. The criteria for classifying consumption as “Adequate” or “Inadequate” must be explicitly defined in the methods section (e.g., based on national dietary guidelines).

Abbreviation: SD, standard deviation.

## 4 Discussion

Mental disorders are a major public health issue due to their high prevalence, social impact, and consequences in the lives of the affected individuals. Among them, depression is one of the most frequent [18, 19]. According to the World Health Organization (WHO), 5% of the world population suffers from depression, which represents more than 350 million people [20].

Our study found a higher prevalence of mental disorders in women (53.4%) compared to men (44.2%), though this difference did not reach statistical significance (Table 1). This trend aligns with prior research indicating that women are more susceptible to depression than men [21, 22]. This directional pattern is consistent with established literature indicating women′s heightened susceptibility to depression [22, 23] though hormonal and genetic mechanisms [24] underlying this disparity require further investigation in the Brazilian context. This study identifies young adults (18–30 years), singles, and individuals with secondary education as high‐risk groups for mental disorders in São Paulo.

The identified triple vulnerability—being young, single, and having average schooling—reflects the intersections between vital development, social organization, and urban inequality. Economic crises and unassisted transitions disproportionately impact young adults; singles suffer from the erosion of protective networks in individualized societies; and high school workers face precariousness without adequate support networks [25]. The pandemic exposed and exacerbated these social fractures, transforming threshold groups into high‐risk populations for mental disorders [26]. The strong association between psychotherapy and mental disorders (*p* < 0.0001) reflects reverse causality bias: symptomatic individuals seek treatment. Although psychotherapy is a therapeutic resource, its association with disorders here indicates that it is predominantly sought after the emergence of symptoms.

Contrary to expectations of homogeneous dietary patterns, we identified significant differences in the consumption of key foods among individuals with and without mental disorders (Table 2). Participants without disorders consumed substantially more vegetables (11.4 vs 7.7 *p* < 0.001)—foods rich in fibers, antioxidants, and micronutrients with neuroprotective properties [27]. Paradoxically, the group with disorders reported higher consumption of fruits (12.3 vs. 5.7, *p* < 0.001) and oils/fats (12.5 vs. 11.4, *p* = 0.03). This fruit‐fat pattern suggests compensatory mechanisms such as the search for fast energy via fruits (especially juices and sugary variants), and hyperpalatability as an emotional regulation strategy. Despite the healthy status of fruits, their excessive consumption can exacerbate metabolic dysregulation in vulnerable populations [12, 27, 28].

Gopinath et al. [12] found in their study that fiber intake and increased consumption of fruits and vegetables were inversely associated with the development of depressive symptoms. Foods with a high glycemic index, as they cause increased inflammation in the body, were present in greater quantities in the diet of people with depressive symptoms. This inflammatory state, generated by the release of cytokines, interferes with the metabolism of neurotransmitters and the action of brain‐derived neurotrophic factor (BDNF) [29].

The inflammatory cascade triggered by high‐glycemic‐index foods, as observed in individuals with depressive symptoms, may impair serotonergic pathways and BDNF signaling [29]. Conversely, diets rich in unsaturated fats (e.g., olive oil) have been associated with neuroprotection and enhanced endothelial function; however, our findings did not distinguish between trans fats and healthier lipid sources. Sánchez‐Villegas et al. [11] emphasize that trans fats, unlike unsaturated fats, exacerbate depression risk, suggesting that dietary quality—not merely fat quantity—mediates mental health outcomes.

Pearson's correlation analysis revealed specific associations between dietary adequacy and mental disorders (Table 3). There is a moderate positive correlation for adequate consumption of oils/fats (*r* = 0.11; *p* < 0.01), suggesting a protective effect of high‐quality lipid sources (e.g., olive oil, nuts). Paradoxically, inadequate meat consumption was associated with higher SRQ‐20 scores (*r* = 0.00; *p* = 0.004), possibly reflecting protein deficiencies or excessive intake of processed foods. Contrary to expectations, legumes (beans, lentils) did not show a significant association (*p* = 0.16), indicating that unmeasured factors, such as the method of preparation, may mediate their impact.

Although we have not dosed biomarkers, the protective correlation of oils/fats aligns with the literature on omega‐3 fatty acids as modulators of anti‐inflammatory cytokines. These findings suggest that both food quality and quantity modulate mental health outcomes, potentially through inflammatory pathways and neurotrophic mechanisms (e.g., BDNF) [29, 30].

The findings also show that lipid quality (adequacy of oils/fats) and protein balance (avoid inadequacy of meat) are critical dietary factors for mental health. At the same time, legumes were neutral in this context. The absence of association with other groups suggests that interventions should prioritize replacing trans fats with unsaturated ones and ensuring access to high‐quality protein sources.

This study has limitations, including its cross‐sectional design, which precludes causal inferences, and reliance on self‐reported dietary data, susceptible to recall bias. The absence of biomarkers (e.g., BDNF, inflammatory cytokines) limits mechanistic insights, and the online recruitment strategy may underrepresent socioeconomically disadvantaged groups. Future longitudinal studies incorporating biochemical assays are needed to elucidate diet‐depression pathways.

Although limited by its cross‐sectional design and reliance on self‐reported data, this study makes a valuable contribution to the understanding of dietary psychiatry in Brazil. This context has been underrepresented in the literature. Future research should integrate biomarkers (e.g., BDNF, cytokines) and longitudinal designs to clarify causal relationships. Clinically, these results advocate for multidisciplinary approaches in mental health care, where nutritionists, psychologists, and public health policymakers collaborate to address diet as a modifiable risk factor for depression.

## 5 Conclusion

This study revealed significant associations between sociodemographic factors, dietary patterns, and common mental disorders among adults in São Paulo during the COVID‐19 pandemic. The findings indicate a higher prevalence of disorders among younger adults (18–30 years), single individuals, those with secondary education, and those receiving mental health care. Regarding dietary patterns, individuals with mental disorders showed a distinct profile characterized by a significantly lower intake of vegetables but a higher consumption of fruits and oils/fats compared to those without disorders. Correlation analyses revealed that a higher consumption of oils and fats was positively associated with SRQ‐20 scores (*r* = 0.11; *p* < 0.01). Although a significant association was found for inadequate meat consumption (*p* = 0.004), the correlation coefficient was negligible (*r* ≈ 0), indicating a statistically significant but likely nonmeaningful effect. These findings highlight the complex relationship between diet and mental health in this population, particularly underscoring the unexpected pattern of fruit consumption, which warrants further investigation.

## Author Contributions

**Jessica Leitão Morilla:** investigation, validation, methodology, visualization, writing – original draft, writing – review and editing, formal analysis, and data curation. **Luiz Henrique da Silva Nali:** investigation, writing – original draft, validation, methodology, and data curation. **Fernanda Simões da Costa Fujino:** investigation, writing – original draft, validation, methodology, visualization, writing – review and editing, and formal analysis. **Daniel Ramos Olcerenko:** visualization, writing, review, and editing. **Patricia Colombo‐Souza:** conceptualization, writing – review and editing, visualization, methodology, validation, project administration, supervision, and resources.

## Consent

Informed consent was obtained from all participants before the study's commencement.

## Conflicts of Interest

The authors declare no conflicts of interest.

## Transparency Statement

The corresponding author, Patrícia Colombo‐Souza, affirms that this manuscript is an honest, accurate, and transparent account of the study being reported; that no important aspects of the study have been omitted; and that any discrepancies from the study as planned (and, if relevant, registered) have been explained.

## Data Availability

The data that support the findings of this study are available from the corresponding author upon reasonable request.
